# Risk Factors and Incidence of Repeat Osteoporotic Fractures Among the Elderly in Taiwan: A Population-based Cohort Study

**DOI:** 10.1097/MD.0000000000000532

**Published:** 2015-02-20

**Authors:** Peng-Ching Hsiao, Tzeng-Ji Chen, Chung-Yi Li, Chi-Ming Chu, Tung-Ping Su, Sheng-Hao Wang, Hsueh-Hsing Pan, Kwua-Yun Wang

**Affiliations:** From the Graduate Institute of Medical Sciences, National Defense Medical Center (PCH, HHP, KYW); Department of Nursing, Tri-Service General Hospital (PCH, HHP); Department of Family Medicine, Taipei Veterans General Hospital, Taipei (T-JC); Department of Public Health, National Cheng Kung University, Tainan (C-YL); Section of Health Informatics, Institute of Public Health, National Defense Medical Center and University (C-MC); Department of Psychiatry, Taipei Veterans General Hospital (T-PS); Department of Orthopedics, Tri-Service General Hospital (SHW); Department of Nursing, Taipei Veterans General Hospital, Taipei, Taiwan (K-YW).

## Abstract

The incidence of osteoporotic fracture (OF), a condition that leads to higher morbidity and mortality in the elderly, is increasing yearly worldwide. However, most studies of OF have focused on the epidemiology of initial fractures, mainly in female and white populations. This study aimed to explore the incidence and the risk factors for repeat osteoporotic fracture (ROF) in Taiwan.

We performed a retrospective cohort study using the Taiwan National Health Insurance Database (NHIRD) from 1995 through 2011. Individuals aged 65 years or older who experienced an initial OF were included. The patients were followed until death, the end of registration in the NHIRD, ROF occurrence, or the end of the study period **(**December 31, 2011**)**, whichever occurred first. The incidence of ROF over ≥5 years after the initial fracture was analyzed, and the risk factors for ROF were assessed using Cox proportional hazards models.

The incidence rates of ROF were 950.5, 321.4, 158.7, 92.8, and 70.2 per 1000 person-years among subjects in their first, second, third, fourth, and fifth years after the initial OF, respectively. Nearly 45% of the subjects sustained a ROF in the first year after OF. ROF risk increased with age and Charlson Comorbidity Index (CCI) score. Greater risk for ROF was observed among female subjects and those who had suffered from hip and vertebral fracture at the first OF, had undergone OF-related surgery, and had received bone-related medications.

The incidence of ROF in the Taiwanese elderly is higher during the first year after the initial OF, and ROF risk increases with age, female sex, high CCI score, and in those who have undergone OF-related surgery, sustained hip or vertebral fracture, and used bone-related medications.

## INTRODUCTION

The aging of the population is an inevitable and predictable worldwide phenomenon,^1^ and Taiwan is no exception. The proportion of Taiwanese elderly over 65 years of age reached 10.7% in 2011 and is expected to rise to 20% in 2025. As a result, Taiwan will become a super-aged society, and its aging rate will be the fastest among developed countries.^2^ Thus, elder health care will pose major challenges in Taiwan. Furthermore, with the population quickly aging and with productivity rapidly declining, people in Taiwan will assume greater medical expenses and costs for long-term care.^2^

Along with an aging population, osteoporosis, which is usually identified as an age-related disease, has rapidly increased and become a widespread public health problem worldwide.^3–5^ Osteoporosis is one of the major causes of fracture and repeat fracture among the elderly.^6^ The incidence of osteoporotic fractures (OF) varies for different fracture locations and in different populations.^3,5,7–9^ However, the number and incidence rates of OF are increasing among the older population yearly, particularly in Asia.^5,8,10^ Moreover, studies have shown that fracture patients, particularly those with osteoporosis, may be more likely to suffer from repeat osteoporotic fractures (ROFs) than those without a history of fracture, and ROF patients have been shown to have increased levels of mortality, morbidity, and dependence in their daily activities.^4,11–14^ Older people with OF, particularly those with hip or vertebral fractures, often present a tendency for depression and disability, and the consequences of OF, such as the length of hospital stay and rates of morbidity and mortality, are usually higher in the elderly than in younger adults.^15–18^ As a result, these elderly individuals may become a long-term burden for family members who serve as day-to-day caregivers and may also constitute a high economic burden to society.^19,20^ However, individuals may not be aware that they are osteoporotic until they sustain their first OF. Consequently, greater attention should be paid to the risk and incidence of ROF to prevent a second fracture.

Furthermore, the majority of epidemiological studies on OF have focused on female or white populations.^3,11,21–23^ Additionally, most studies have investigated a single fracture location, particularly the hip.^8,13,16,18,23,24^ Thus, little is known about the incidence and risk factors of other fracture locations for ROF in the non-white population. Moreover, some previous studies suffered from limitations such as a small sample size, a short follow-up period, or the selection of only inpatients as study subjects.^16,24–26^ Therefore, the current study aimed to investigate the incidence and risk factors of ROF among the elderly, of both sexes, in Taiwan. The results of this study may assist health practitioners in designing appropriate interventions to reduce ROF among the elderly, in allowing the elderly to age actively, and in limiting fracture-related healthcare costs in the future.

## MATERIALS AND METHODS

### Data Source

Data sets were obtained from Taiwan's National Health Insurance Research Database (NHIRD). Taiwan has introduced a single-payer National Health Insurance program (NHI) in 1995, and 99% of the population was enrolled and is still currently enrolled in the insurance program.^27^ The NHIRD contains the demographic data of enrollees; service records and expenditure claims from outpatient, inpatient, and ambulatory care; and data associated with contracted pharmacies for reimbursement purposes. However, information on patient lifestyle (smoking, alcoholism, body mass index [BMI], and caffeine use), examination data (x-rays, bone mineral density [BMD]) and the use of self-paid medications (vitamin D, calcium) is not available in the NHIRD. The National Health Research Institutes, the only institution approved for conducting sampling processes in Taiwan, creates a random sample of 1,000,000 people from a population of 21,400,826 enrollees throughout Taiwan each year for academic research purposes. The samples in the NHIRD are representative of the population in Taiwan. The National Health Research Institutes encrypts the data of any information that might identify a specific patient, and data confidentiality is guaranteed based on the data regulations specified by the National Health Research Institutes.^27^ This study was approved by the Institutional Review Board in Taiwan (IRB NO. VGHIRB 2014–06–003CE), which waived the requirement of informed consent for this population-based cohort study.

### Selection of Cases

**FIGURE 1:**
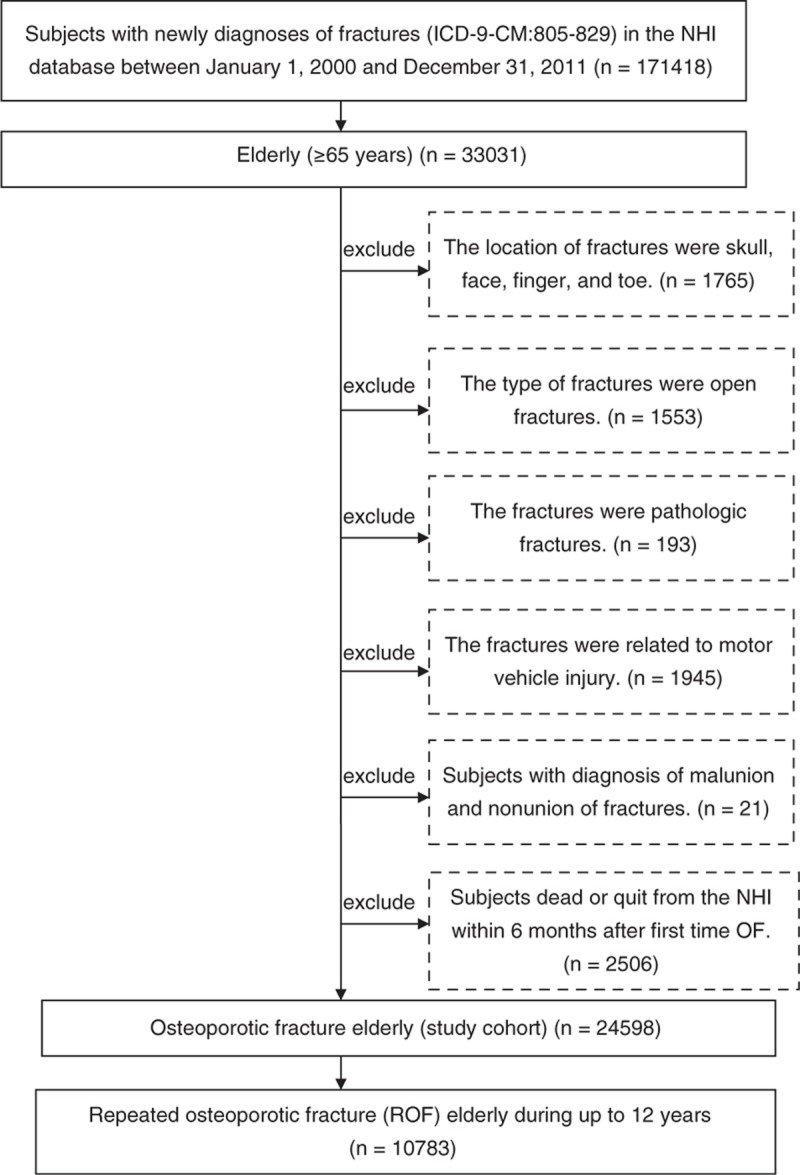
Subject selection flow chart.

This was a retrospective cohort study. We used the NHI program database of 1,000,000 NHI beneficiaries for the years 1995 to 2011. Patients aged 65 years and older who had a new study-defined OF diagnosis based on the International Classification of Diseases, 9th revision, Clinical Modifications (ICD-9-CM) between January 1, 2000 and December 31, 2011 constituted the study cohort. A “new” fracture (ICD9 code 805–829) was defined as the first diagnosis of the fracture at an ambulatory visit or during a hospital stay within the index period, with no coded fracture of the same body part in the previous 5 years. “Study-defined” OF included any fracture except those on the skull, face, finger, or toe.^21^ Open fractures and motor vehicle injuries that were suggestive of high force, pathologic fracture (ICD9 code 733.1), or fracture malunion and nonunion (ICD9 code 733.8) were excluded. Our study-defined OF cases were associated with decreased bone mass, whereas the excluded fractures were not.^21^ We also excluded subjects who died or who separated from the NHI within 6 months after the first OF. We identified 24 598 elderly patients with newly diagnosed OF during the study period as the study cohort. The study cohort was followed until death, the end of registration in the NHIRD, the occurrence of ROF, or the end of the study period (December 31, 2011), whichever occurred first. The ROF participants were subjects who sustained study-defined OF 6 months after the index date, yielding a total of 10 783 eligible participants during the study period (Figure 1). The index date for each case was the date of his/her first-time hospitalization or the date that the subject received outpatient services for OF during the study period. We chose a 6-month period to allow a reasonable amount of time for fracture healing to have occurred and thereby to increase the chances that a coded ROF was not an unhealed initial fracture.

### Study Measures

The primary outcome of our study was the incidence of ROF, whereas the secondary outcome was ROF risk factors. The participant demographic data, including age, sex, and low-income households, were identified at the index date. Data regarding fracture-related surgery (ICD 9 codes 78.1, 78.5, 79, 78.4, 78.9, and 81), the fracture location, and comorbid conditions that might increase fracture risk were also collected. The level of comorbid conditions was calculated using the Charlson Comorbidity Index (CCI).^28^ Additionally, we examined the use of medications associated with the development of osteoporosis. We defined the use of medications associated with the development of osteoporosis as any instance in which anticonvulsants (phenobarbital, phenytoin, carbamazepine), corticosteroids, thiazolidinedione, proton-pump inhibitors (omeprazole, lansoprazole, pantoprazole, rabeprazole, esomeprazole), warfarin, or heparin had been chronically dispensed at the appropriate dosage per day for >90 days, but within 6 months after the index date to observe sufficient effects from the medications.^21^ The medications related to OF were retrieved from the medication history during the study period and were selected using the World Health Organization Anatomical Therapeutic Chemical classification system.

### Statistical Analyses

Continuous variables were descriptively expressed as the mean ± standard deviation and as proportions for categorical variables. We used person-time incidence rates (per 1000 person-years) to represent the ROF incidence rate. The differences in the cumulative ROF incidence in different demographic categories and ROF risk factors were analyzed using Cox proportional hazard models. Microsoft's SQL Server 2005 (Microsoft Corp, Redmond, WA) was used for the data extraction, linkage, processing, and sampling. All statistical analyses were performed using SPSS statistical software (version 20.0 for Windows; IBM Corp, New York, NY). Statistical significance was defined as a *P* value <0.05.

## RESULTS

### Characteristics of the Study Cohort

The mean age of the elderly individuals with first-time OF was 75.3 ± 6.9 years. A preponderance of female subjects was observed in the study cohort. The vertebral column was the most common location for OF occurrence in the Taiwan elderly. The surgical rate among elderly subjects who sustained first-time OF was 28.0%. The proportion of OF among the elderly from low-income households was low. Elderly individuals with a CCI score ≥3 and those who chronically used bone-related medications (medications associated with the development of osteoporosis) were more likely to sustain OF (Table 1).

**TABLE 1:**
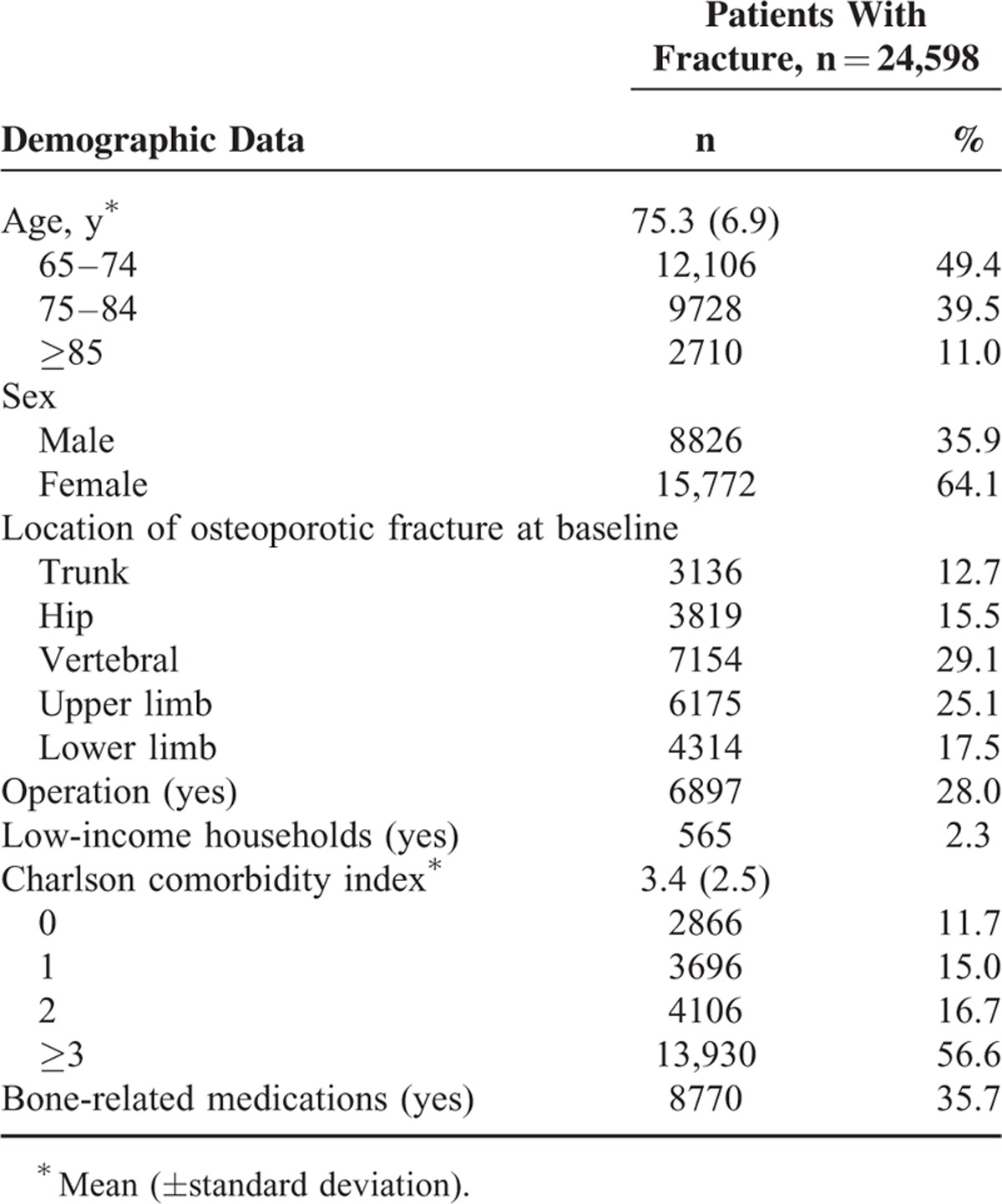
Baseline Characteristics of Subjects With First-time Osteoporotic Fracture

### The Incidence of ROF

Table 2 shows the incidence of ROF in patients with first-time OF. Of the 24 598 subjects, 10 783 subjects sustained ROF during the study period (Figure 1). The ROF incidence in the first year after first-time OF was 950.5 per 1000 person-years, which was significantly higher than the other time periods after the initial OF. Nearly 45% of subjects sustained ROF within 1 year after their first OF. The ROF incidence rates for subjects with osteoporosis were 321.4, 158.7, 92.8, and 70.2 per 1000 person-years during the second, third, fourth, and fifth years after the initial OF, respectively. Furthermore, older age was associated with a higher incidence of ROF, and the incidence of ROF in females was higher than that in males. The most common ROF locations were the hip and vertebral column. Elderly individuals who underwent OF-related surgery had a CCI score ≥3, were chronic users of bone-related medications, and showed higher incidence rates of ROF.

**TABLE 2:**
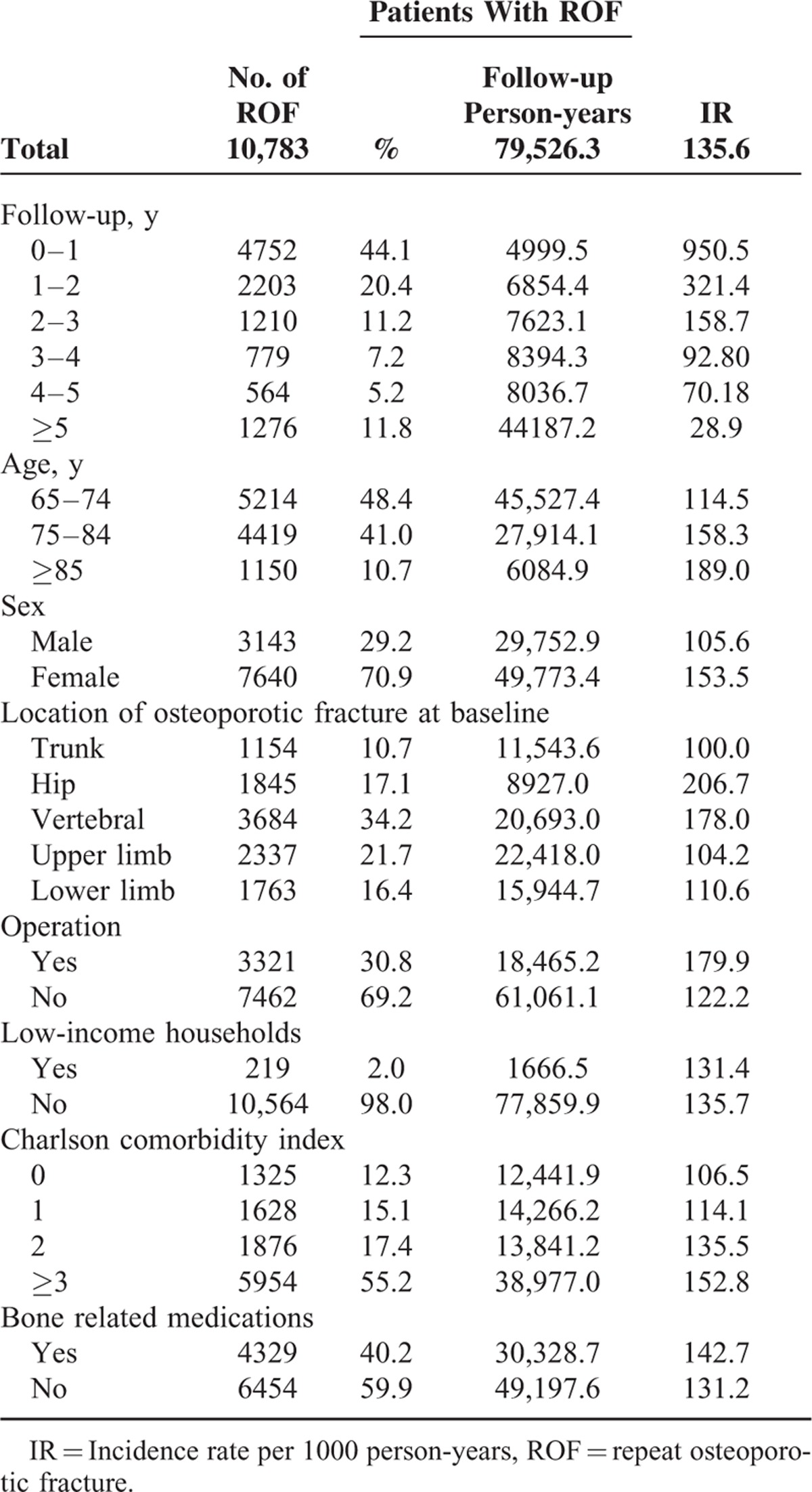
Incidence of ROF in Patients With First-time Osteoporotic Fracture

### The Risk Factors for ROF

**FIGURE 2:**
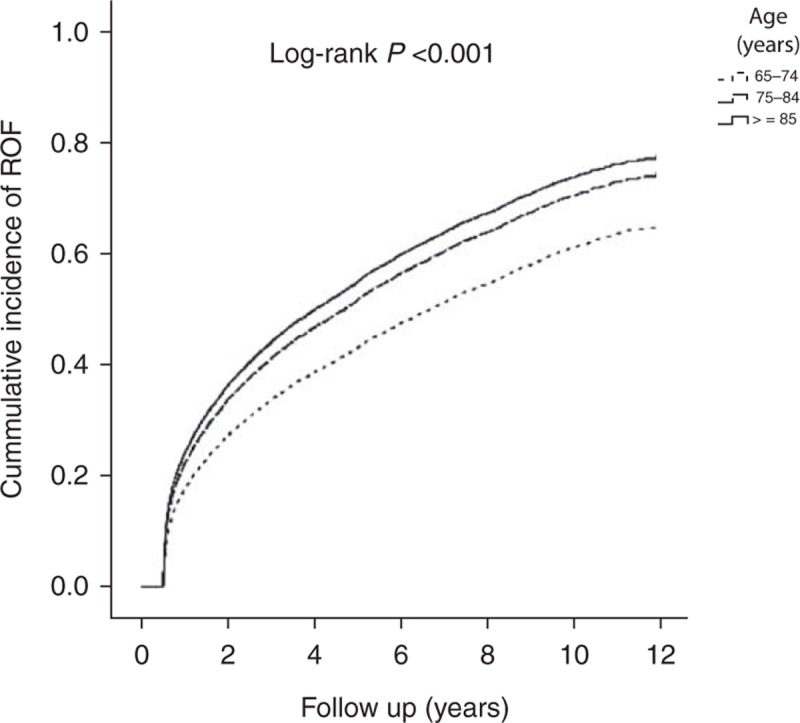
Cumulative incidence of ROF among different age groups. OF = osteoporotic fracture, ROF = repeat osteoporotic fracture.

**FIGURE 3:**
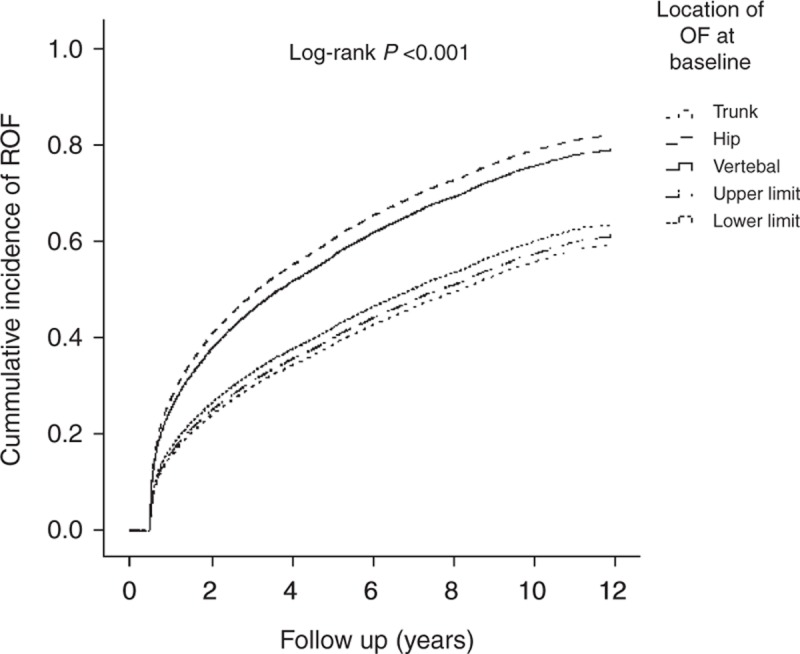
Cumulative incidence of ROF among different OF locations at the baseline. OF = osteoporotic fracture, ROF = repeat osteoporotic fracture.

**FIGURE 4:**
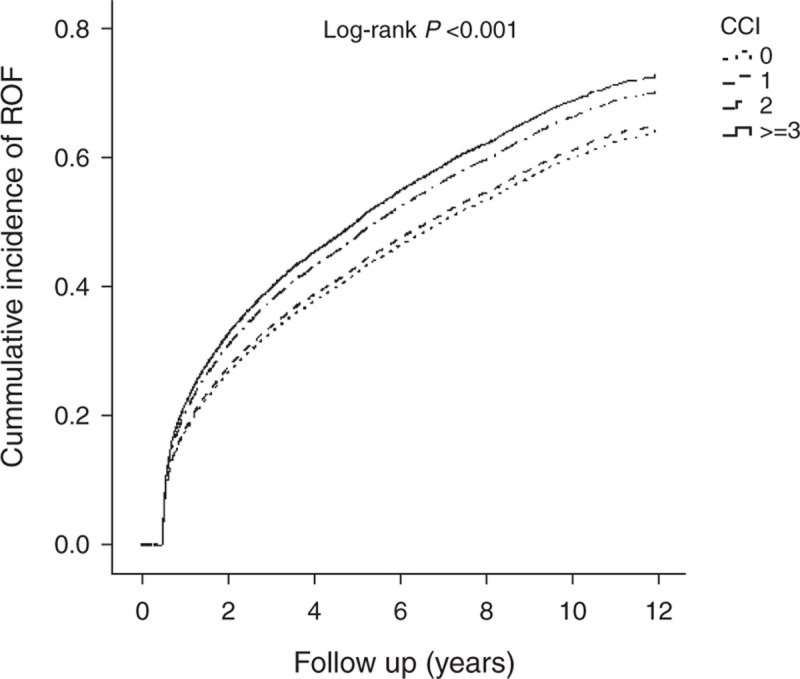
Cumulative incidence of ROF among different Charlson comorbidity indices. ROF = repeat osteoporotic fracture.

Table 3 shows the analyses of the predictive factors for ROF in subjects with first-time OF. Factors with *P* values ≦0.05 in the Cox univariate proportional hazard model were entered into the Cox multivariate proportional hazard model to ascertain the hazard ratio (HR) of each factor and the risk factors for ROF. Elderly individuals aged ≥85 years showed a higher ROF risk than subjects between 65 and74 years of age (HR = 1.26, 95% confidence interval [CI] 1.18–1.34, *P* < 0.001). Figure 2 shows the Kaplan–Meier estimates of the cumulative overall ROF incidence among different age groups. The cumulative ROF incidence rates significantly differed among the various age groups (log-rank *P* < 0.001). Females were more likely to sustain ROF than males (HR = 1.41, 95% CI 1.35–1.47, *P* < 0.001). The vertebrae and the hip were the most common ROF locations, and the risk of fracture of the vertebrae and hip was higher than that of the trunk, with ROF HRs of 1.59 (95% CI 1.49–1.70, *P* < 0.001) and 1.45 (95% CI 1.34–1.57, *P* < 0.001), respectively. Figure 3 shows the Kaplan–Meier estimates of the cumulative overall ROF incidence among the different OF locations. The cumulative ROF incidence rates significantly differed among the various OF locations (log-rank *P* < 0.001). Moreover, elderly subjects who underwent OF-related surgery and were chronic users of bone-related medications were more likely to sustain ROF, with HRs of 1.35 (95% CI 1.29–1.41, *P* < 0.001) and 1.11 (95% CI 1.07–1.15, *P* < 0.001), respectively. Elderly subjects with a CCI score ≥3 were also more likely to sustain ROF than subjects with a CCI score = 0 (HR = 1.19, 95% CI 1.12–1.26, *P* < 0.001). Figure 4 shows the Kaplan–Meier estimates of the cumulative overall ROF incidence for different values of the CCI. The cumulative ROF incidence rates significantly differed among the various CCI groups (log-rank *P* < 0.001).

**TABLE 3:**
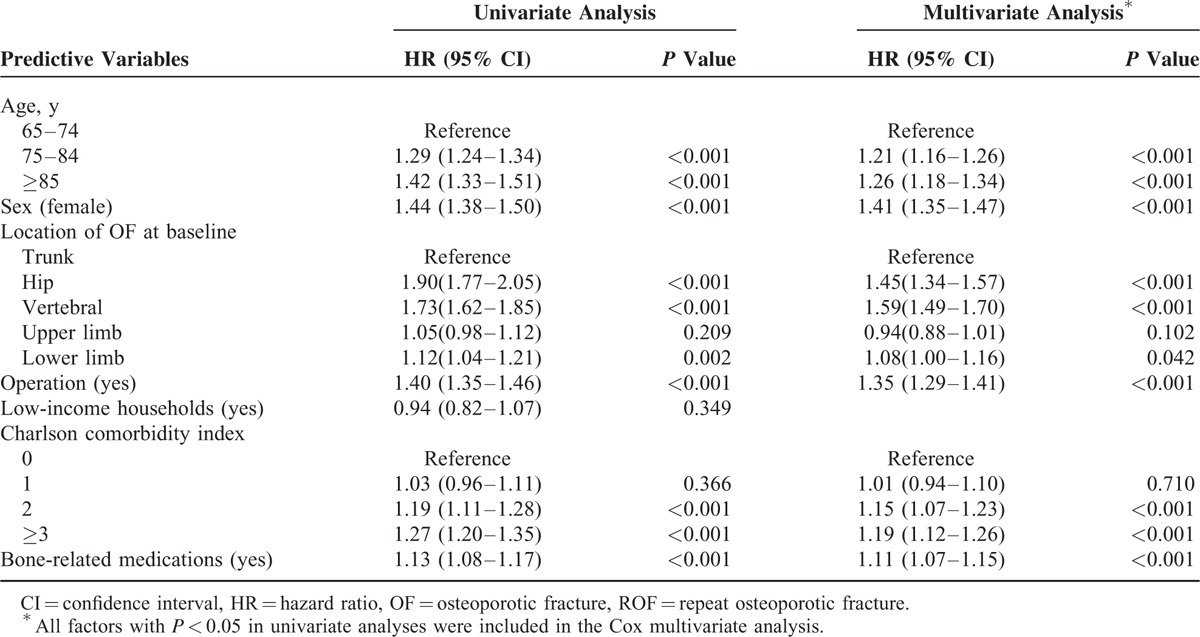
Analyses of Predictive Factors for ROF in Subjects With First-time OF

## DISCUSSION

In our study, we found that the ROF incidence rates were highest in the first year after the initial OF and then declined yearly. Nearly 45% of subjects sustained ROF within 1 year after their first OF. These data are similar to those of Center et al (2007), who indicated that approximately 41% of refractures in women and 52% of refractures in men occurred during the first 2 years after initial fracture.^14^ However, the study results of Ruan et al^25^ showed that the second fracture occurred 3.7 years after the first fracture, on average, and that the refracture rate was 2.12% within 1 year and 4.66% within 2 years. Another study showed that within 5 years following the initial fracture, 24% of women and 20% of men refractured.^11^ The reasons for these differences might be related to the relatively small sample sizes, the relatively younger subjects, and the different ethnic groups included in the studies. Ruan et al^25^ only examined 225 subjects who were older than 50 years, with a follow-up period of 2 years, and Bliuc et al only included 1295 Australians who were older than 60 years. In contrast, our study included 10 783 subjects who were older than 65 years. Most studies have shown that bone density decreases with aging and that the risk of fracture increases when the bone density decreases,^29,30^ which may also have contributed to the different study results. Additionally, methodological differences in sampling and defining and ascertaining ROF may have led to differences in the interpretation of these previous data. Our results may be explained by the fact that the initial OF limits the mobility of the elderly and their performance of daily activities; then, because of these limitations, they may fall easily and sustain repeated fractures. Moreover, the literature has shown that the incidence of refracture varies widely throughout the world.^10^ Our results are consistent with the results of Bliuc et al^11^ (2013) who demonstrated that the long-term refracture rate was reduced, particularly in the elderly, as a result of their high mortality rate. Thus, greater risk reduction intervention or refracture prevention policies are needed, particularly in the first year after initial fracture.

Our data indicate that ROF risk increased with age, CCI score, and OF-related surgery. Research has shown that the incidence rates of refracture increase with aging worldwide, regardless of ethnicity.^5^ High CCI scores and OF-related surgery are indicative of individuals with poorer physiological capacity and a high probability of functional decline, as well as problematic mobility. Thus, such individuals show higher risks for falls and refractures. Li et al (2012) also indicated that most subsequent fractures occurred at the adjacent level within 1 year and that OF-related surgery, such as vertebroplasty, may contribute to the risk of subsequent adjacent fracture.^31^

Our study demonstrated that hip fracture and vertebral fracture represent risk factors for ROF. Chen et al^15^ (2013) indicated that osteoporotic vertebral fractures increase the risk of cardiovascular diseases and stroke. Furthermore, studies have also shown that older people with OF, particularly hip or vertebral fractures, often represent a tendency toward disability and that the consequences of OF, such as the length of hospital stay and high rates of morbidity, are usually greater among the elderly than in younger adults.^15–18^ Hip fracture and vertebral fracture often lead to disability and functional decline to the elderly; thus, elderly individuals with hip fractures or vertebral fractures may fall more easily than those with other fractures and show a higher likelihood of refracture.

Our data also revealed that ROF risk was related to the use of bone-related medications. These results are consistent with the results of other studies, which showed that the chronic use of anticonvulsants, corticosteroids, thiazolidinedione, proton-pump inhibitors, warfarin, or heparin may increase the risk of developing osteoporosis as well as OF.^21,32^ We defined the use of medications associated with the development of osteoporosis as any instance in which anticonvulsants, corticosteroids, thiazolidinedione, proton-pump inhibitors, warfarin, or heparin had been chronically dispensed at the appropriate dosage per day for >90 days, but within 6 months of the index date to observe sufficient effects from the medication. However, understanding dose-related effects or the types of bone-related medication with the greatest impact on ROF requires further investigation.

Our study also demonstrated that females were at a higher risk for sustaining ROF. Another study demonstrated that regardless of race, country or age, the osteoporosis fracture rates, particularly those for hip fracture, were greater in women than men.^5^ The literature indicates that BMD declines more rapidly in women than in men with aging, especially after menopause, and that physical activity is positively associated with BMD. Additionally, lower levels of physical activity have been associated with increased fracture risk,^6,29,30^ and females are therefore at a higher risk of sustaining ROF.

The results of the present study offer an important contribution to the literature, as our findings show that the incidence of ROF in elderly Taiwanese is higher in the first year after initial OF and that ROF risk increases with age, female sex, high CCI score, OF-related surgery, hip or vertebral fracture, and the use of bone-related medications. The risk factors of ROF are nearly the same as those for initial OF in elderly Taiwanese. Recently, Ruan et al^25^ (2011) also indicated that aging, female sex, and prior vertebral fractures or prior hip fractures were risk factors for ROF. Regarding our data, when those factors that showed significance in the Cox univariate analysis were entered into the Cox multivariate analysis, all factors still exhibited strong statistical significance. Thus, the factors identified as having a strong impact on the risk of ROF in Taiwanese elderly included age, female sex, high CCI score, OF-related surgery, hip or vertebral fracture, and the use of bone-related medications. Directing attention toward the risk of ROF is important to prevent a second fracture, particularly in the first year after the first OF, and there is often adequate time between the first and second fractures for interventions to reduce the risk of refracture, particularly for older women with a vertebral or hip fracture. Medication and prevention training are also helpful, and further policies or interventions to reduce ROF risk are worth pursuing in Taiwan.

This study had several limitations. First, information on the subjects’ lifestyles (smoking, alcoholism, BMI, and caffeine use), examination data (BMD), and the use of self-paid medications (vitamin D, calcium) was not available in the NHIRD, which may have somewhat restricted the interpretation of our results. The impact of smoking, alcoholism, BMI, BMD, and the consumption of caffeine, vitamin D, or calcium on the incidence of ROF requires further investigation. Regarding possible future studies, we may develop a ROF-related knowledge educational project for the elderly and examine the program's effects on ROF-related knowledge and refracture prevalence in the elderly. This intervention may also include health information provided to the subjects and educational intervention directed at primary care providers. Moreover, the application of our results in the context of another country is unknown. Thus, further studies are necessary to verify these results in different populations.

## CONCLUSION

In conclusion, the results of the present study suggest that the incidence of ROF in elderly Taiwanese is higher during the first year after the initial OF and that the ROF risk increases with age, female sex, high CCI score, OF-related surgery, hip or vertebral fracture, and the use of bone-related medications. The results of this study may assist health practitioners in designing appropriate interventions to reduce ROF in the elderly, in allowing such individuals to age actively, and in limiting related healthcare costs in the future. It may be helpful to implement ROF risk reduction interventions as soon as possible after initial OF, particularly in older women with hip or vertebral fracture and in those individuals with high CCI scores, recipients of OF-related surgery, and those who have used bone-related medications. However, a larger, multi-institutional prospective study is needed to further support the findings of the current study.
